# Development of Secondary Woodland in Oak Wood Pastures Reduces the Richness of Rare Epiphytic Lichens

**DOI:** 10.1371/journal.pone.0024675

**Published:** 2011-09-22

**Authors:** Heidi Paltto, Anna Nordberg, Björn Nordén, Tord Snäll

**Affiliations:** 1 Department of Ecology, Swedish University of Agricultural Sciences, Uppsala, Sweden; 2 Department of Plant and Environmental Sciences, Gothenburg University, Gothenburg, Sweden; University Copenhagen, Denmark

## Abstract

Wooded pastures with ancient trees were formerly abundant throughout Europe, but during the last century, grazing has largely been abandoned often resulting in dense forests. Ancient trees constitute habitat for many declining and threatened species, but the effects of secondary woodland on the biodiversity associated with these trees are largely unknown. We tested for difference in species richness, occurrence, and abundance of a set of nationally and regionally red-listed epiphytic lichens between ancient oaks located in secondary woodland and ancient oaks located in open conditions. We refined the test of the effect of secondary woodland by also including other explanatory variables. Species occurrence and abundance were modelled jointly using overdispersed zero-inflated Poisson models. The richness of the red-listed lichens on ancient oaks in secondary woodland was half of that compared with oaks growing in open conditions. The species-level analyses revealed that this was mainly the result of lower occupancy of two of the study species. The tree-level abundance of one species was also lower in secondary woodland. Potential explanations for this pattern are that the study lichens are adapted to desiccating conditions enhancing their population persistence by low competition or that open, windy conditions enhance their colonisation rate. This means that the development of secondary woodland is a threat to red-listed epiphytic lichens. We therefore suggest that woody vegetation is cleared and grazing resumed in abandoned oak pastures. Importantly, this will also benefit the vitality of the oaks.

## Introduction

Scattered, very old and large trees in agricultural landscapes are critical habitats for many species and provide a range of ecosystem services [1]. Many such trees, ancient trees henceforth, are situated in wood pastures that are increasingly abandoned due to land use change [2], [3]. If the areas are not converted to other land use, e.g. arable land, whereby the trees are cut down, the open wood pastures naturally develop into secondary woodland. This process is known to reduce the grassland biodiversity [3], but its effects on the biodiversity associated with the remaining ancient trees are largely unknown.

In northern Europe, oaks (*Quercus robur* L. and *Q. petraea* (Matt.) Liebl.) can become older (>900 years) [4] and bigger (>4.5 m in diameter; [5]) than most other tree species. The oldest oaks are probably the most species rich since colonisations accumulate with increasing time [6], [7], and because they provide a broad range of microhabitats for many organism groups (e.g. rougher bark and larger cavities than other trees). Examples of organism groups associated with oaks include lichens [8], [9], bryophytes [8], [10], fungi [11], beetles [12], pseudoscorpions [13], moths [14], birds [15], and bats [16]. Old oaks support a unique epiphytic lichen flora consisting of about 303 species in Great Britain [8] and 140 species in Sweden [17].

The diversity of epiphytic lichens and other organisms on oaks has decreased in Northern Europe and elsewhere. For example, some 20 lichens which are confined to oak are currently in the Swedish Red Data Book [18]. Of these, 13 species are typical of ancient oak trees in sun-exposed conditions [19], [20]. The historical felling of oaks [21]–[22] and woodland succession following abandonment of grazing are two possible explanations for this decline. The distribution of red-listed and other rare epiphytic lichens on ancient oaks at a regional spatial scale suggest an effect of habitat loss on these species [11], [23], and many ecologists and nature conservationists have suggested that increased shade as a consequence of the development of secondary woodland negatively affects lichens of conservation concern, e.g. [8], [24]. We are, however, not aware of any scientific studies exploring the effect of secondary woodland. Several large-scale biodiversity restoration programs are either underway or being planned in oak wood pastures in Sweden and elsewhere in Europe. This also increases the need for robust evaluation of the effect of the development of secondary woodlands on the species associated with old oaks.

The general aim of this study is to investigate the effect of the development of secondary woodland in oak wood pastures on national and regional red-listed epiphytic lichens on ancient oaks. Specifically, we tested for differences in species richness, and for differences in occurrence and abundance of a set of lichen species between 1) ancient oaks in secondary woodland and 2) ancient oaks in open conditions. We refined the test of the effect of secondary woodland by also including other variables in the analyses: canopy cover, bark pH, abundance of bryophytes, depth of bark crevices, and density of oaks in the surrounding landscape.

## Methods

### Study region

**Figure 1:**
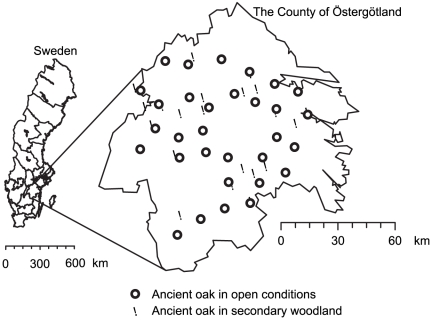
The location of the study oaks.

We chose the County of Östergötland in Sweden as the study region (Fig. 1) because of its high density of oak [21]. This region is nationally and internationally important for the conservation of organisms associated with oak [21], [25], with 18,000 ha of oak environments of high value for conservation (1.7% of the land area in the county). Oaks were abundant in prehistoric times [26], but many were cut down during the 16^th^ and 19^th^ centuries [22]. During the last 80 years, the standing volume of oaks [27], and in particular the number of medium-sized and large oaks (>35 cm; unpublished data, Johan Bergstedt), has increased in Sweden. Data, however, on the change in the number of oaks larger than 100 cm in diameter is not available.

### Selection of study oaks in open conditions and in secondary woodland

Between 1997 and 2008 all large trees (≥100 cm in diameter at breast height) in the county were mapped [28] (http://www.tradportalen.se/). In this large scale survey, trees were recorded as being located in open, semi-open, or shady conditions. For the current study we selected 21 oaks located in shady conditions and separated by at least 6 km. Thereafter we selected oaks located in open conditions studied in Paltto et al. [23]. The selection criterion was to include all oaks from Paltto et al. [23] that were located less than 15 km from the oaks located in shady conditions. We chose this short arbitrary distance to minimize the risk of different connectivity surrounding ancient oaks between study oaks in open conditions and in secondary woodland, since this connectivity explains the occurrence of our study species [23]. The diameters at breast height were selected to be 120–140 cm, and the study trees can hence be considered to be “ancient”. The study oaks in secondary woodland were surrounded by trees with a diameter of 10–30 cm (approximately 10–50 years) within a radius of 10 m. More than half of the study oaks in secondary woodland were in direct contact with leaves or needles from the surrounding trees. The study oaks in open conditions were not surrounded by such trees within 10 m, and about half of these oaks were not surrounded by any trees within a radius of 50 m. The trees consisted of silver birch (*Betula pendula* Roth), norway spruce (*Picea abies*), aspen (*Populus tremula* L.), oaks (*Q. robur/Q. petraea*), norway maple (*Acer platanoides* L.), rowan (*Sorbus aucuparia* L.), swedish whitebeam (*Sorbus intermedia* (Ehrh.) Pers.), scots pine (*Pinus sylvestris* L.) and hazel (*Corylus avellana* L.). Five out of the 21 trees in the secondary woodland were surrounded by coniferous norway spruces, or a few scots pines. All trees were growing at least 80 m from main roads, and at least 20 m from minor roads, arable fields or water to avoid confounding the results with the impacts from air pollution, dust or contrasting microclimates.

### Study species and environmental variables

On each of the 52 study oaks, we surveyed ten lichen species (Table 1, for species characteristics see Table S1). These species were the result of the following selection criteria (as in [23]): they should be possible to determine in the field from the ground; they should be red-listed according to the IUCN criteria [18] (eight species) or according to the regional list by Ek et al. [29] (two species); and they should be facultatively associated with ancient oaks (Table S1). Occasionally, some of them grow on other tree species or younger oaks, especially in areas with a high density of old oaks [30]. Nine of the species are crustose and one is foliose.

**Table 1:** Red-list category, number of trees occupied and abundance of the study lichens on 52 ancient oaks in open conditions or in secondary woodland.

		Number of occupied trees		Abundance (min and max no of grid cells)	
Species	Red-list category*	Secondary woodland	Open conditions	Secondary woodland	Open conditions
*Chaenotheca phaeocephala*	regionally	10 (48%)	24 (77%)	1–53	7–111
*Cliostomum corrugatum*	NT	2 (10%)	11 (26%)	15–44	1–94
*Buellia violaceofusca*	NT	3 (14%)	8 (26%)	7–12	2–47
*Ramalina baltica*	NT	0	7 (23%)		1–78
*Calicium adspersum*	regionally	3 (14%)	4 (13%)	14–17	1–55
*Schismatomma pericleum*	NT	1 (5%)	2 (6%)	1–1	2–11
*Calicium quercinum*	VU	0	2 (6%)		3–5
*Caloplaca lucifuga*	NT	0	2 (6%)		3–5
*Sclerophora coniophaea*	NT	2 (10%)	0	1–4	
*Lecanographa amylacea*	VU	0	1 (3%)		2

*Nationally red-listed according to IUCN (VU = vulnerable, NT = near threatened [18]), or regionally red-listed [29]).

We surveyed the abundance of each species using a soccer goal net (grid cell size 13 cm×13 cm) that was attached around the oak trunk at 60–160 cm above the ground. We used the number of grid cells with a species present as the measure of species abundance. Total number of cells (excluding a small number of cells covering decorticated trunk) represented our sampling effort (offset variable; see *Statistical modelling* below). The average number of cells per tree was 292 (range: 258–483). Species richness is defined as the total number of species out of the ten species surveyed. The field work was conducted in June and July 2007.

The main explanatory (treatment) variable was whether the oak was located in open conditions or in secondary woodland, a variable that is easy to alter by practical management. In addition, we included five nuisance explanatory variables that are known to explain the distribution of epiphytes, with the aim of extracting the main effect of open conditions versus secondary woodland.

The first nuisance variable was canopy cover, i.e. the cover of branches and leaves on the focal oak (the main influence) and of trunks, branches and leaves on the trees surrounding the focal oak. The measure reflects the current degree of shade on the bark. Canopy cover was measured by standing with your back against the trunk of the focal oak and looking through a square lattice at arm's length. The lattice frame was 50 cm×50 cm, with a grid cell size of 10 cm×10 cm. Each grid cell was categorised as either “light” (0% canopy cover), “mixed” (1–90% canopy cover) or “shaded” (91–100% canopy cover). The lattice was moved to cover every part of the sky (corresponding to a half sphere) from four positions around the trunk. Each grid cell was assigned one of three values corresponding to the mean of the canopy cover category (0%, 45% or 95%), and the tree-specific canopy cover constituted the mean of all grid cell values. The second variable was the maximum bark crevice depth which was measured using a ruler. Thirdly, we measured bark pH. Bark samples were collected at 1–1.5 m height at four points around the tree trunk. The flakes of bark collected (max. 3 mm thick) were dried in 70°C for 72 hours, and lichens, bryophytes and fungal fruiting bodies were then removed. Next, the pieces of bark were milled and 0.5 g of the powder was blended with 5 ml of deionised water and shaken for 12 hours. The samples were then centrifuged and pH was measured in the clear phase. Fourthly, we quantified the total abundance of bryophytes using the same method as for lichens. This variable was a proxy for competition or local bark moisture. Finally, we used landscape scale variables explaining occurrences of red-listed lichens [23], in accordance with metapopulation and landscape ecology theory [31], [32]. Specifically, we included the variables that explained most of the variation according to Paltto et al. [23]: In the models for species richness, *Cliostomum corrugatum*, *Buellia violaceofusca* and *Calicium adspersum* we included density of oaks >160 cm in diameter at breast height within 0.5 km from each study oak; and for *C. phaeocephala* we included density of oaks >100 cm in diameter at breast height within 5 km.

Raw data on species and environmental variables are given in Table S2.

### Statistical modelling

We modelled the three response variables species richness, occurrence and abundance using the generalised linear modelling framework [33]. For species richness we fitted Poisson models and used a logarithmic link function. For individual lichen species we fitted zero-inflated regression models and hence, jointly modelled the abundance and occurrence [34], [35]. Zero-inflated models are two-component mixture models which include a count sub-model analysing the relationship between abundance and explanatory variables, and a binomial sub-model analysing the relationship between non-occurrence and the explanatory variables. They are appropriate for count data with an excess number of zeroes in comparison with what is assumed by the Poisson distribution [35], [36]; the individual species modelled were absent from at least 30% of the study trees (Table 1). For the count sub-model we used a negative binomial error distribution (due to Poisson overdispersion) and a logarithmic link function, and for the binomial sub-model we used a binomial error distribution and a logit link function. In all count (sub-)models (species richness and local abundance), we accounted for varying sampling effort on different trees by including the number of grid cells surveyed as an offset variable.

A species absence or zero abundance on a tree may arise for three reasons: 1) no diaspore have reached the tree, 2) no diaspore have established (both are also possible after a local extinction), or 3) the local population is outside the sampling grid on the tree. A classic Poisson model for species abundance is inappropriate because of 1) or 2), and a binary presence-absence model for species occurrence is inappropriate because of 3). In applying the zero-inflated model we assume thus, that the excess zeroes of the Poisson distributed abundance are due to 1) or 2).

For species richness, we report parameter estimates of a multi-model which averages over all plausible models. This model building started with identifying the plausible models. The selection criterion was Akaike's Information Criterion for small sample sizes (AIC_c_). AIC_c_ is a measure of relative model fit, and is proportional to the likelihood of the model penalized for the number of model parameters [37], [38]. The definition of plausible model was that it had “substantial empirical support” sensu [38], i.e. that its AIC_c_ was less than two units higher than AIC_c_ for the model with the lowest AIC_c_ (ΔAIC_c_<2.0). Next, we calculated the Akaike weight of each model. The Akaike weight of a model is essentially its probability compared to the probability of the other plausible models, and the sum of the Akaike weights for all plausible models is 1. Finally, we fitted the multi-model, where the estimate of a focal parameter is a weighted average of the estimates of the focal parameter in the plausible models. The weight of the parameter estimates of a model is proportional to the model's Akaike weight [38]. The estimates of parameters that are not included in all plausible models are set to zero in models where these parameters are not included.

For individual species, we started the model building by investigating which single explanatory variables and biologically reasonable two-way interactions led to decreased AIC_c_ when included in the model. Next, we built multiple models with these variables and interaction terms. The models were manually simplified based on AIC_c_ [38]. We do not present multi-models for individual species because we are not aware of software for averaging over zero-inflated regression models. However, since the variables secondary woodland, canopy cover and bryophyte abundance were correlated, we tested replacing these variables with one another in the final models. We present the models with the lowest AIC_c_ in the main article, and other plausible models (ΔAIC_c_<2.0) in Table S3.

We applied the following restrictions in the model building for individual species. First, they should occur on more than five trees. For the infrequent *C. adspersum* (seven occupied trees) we included a maximum of one explanatory variable, and for *C. corrugatum* and *B. violaceofusca* we included a maximum of two explanatory variables in the count sub-models (13 and 11 trees occupied, respectively). The number of ancient oaks in the landscape was not included in the species abundance models because of a likely negligible effect. *R. baltica* only occurred on oaks in open conditions and we therefore tested for difference in occurrence between secondary woodland and open conditions using Fisher's exact test.

Before model building, all explanatory variables were centred (subtracted with their mean) to avoid potential misinterpretation of the main effects of variables that are also part of interaction terms [39]. The parameter estimates and confidence limits of the models for species richness and the sub-model for abundance were transformed as E_t_ = exp(E_m_), where E_m_ is the original estimate. E_t_ expresses the proportional change in species richness per unit change in the predictor variable. Statistical modelling and testing was performed using the software R 2.12.1 (The R foundation for statistical computing, 2010), with the add-on library “pscl” version 1.03.6.1 for fitting zero-inflated models [35], and the add-on library MuMIn (multi-model inference) for Poisson model averaging.

## Results

The ten lichen species were found on 1–34 (2%–65%) out of the 52 ancient oaks surveyed (Table 1). The species richness per oak ranged from 0–5 (mean = 1.6; median = 1).

The canopy cover ranged from 55%–86% among the oaks in secondary woodland (n = 21), and 45%–75% among the oaks in open conditions (n = 31; two-tailed permutation test of the difference: p = 0.001). There was low co-variation between pair-wise combinations of explanatory variables (Table 2; Table S4).

**Table 2:** Characteristics of variables included in regression models explaining richness, occurrence and abundance of red-listed lichens on 52 ancient oaks.

	Oaks in secondary woodland (n = 31)				Oaks in open conditions (n = 21)			
	Median	Average±SD	Min	Max	Median	Average±SD	Min	Max
**Local variables**								
Canopy cover (%)	54	56±8	45	75	69	70±9	55	86
Bryophyte abundance	19	18±11	0	50	31	33±20	1	71
(% of grid cells)								
Bark pH	4.6	4.7±0.6	3.7	6.8	4.6	4.7±0.5	3.6	5.4
Max bark crevice depth (mm)	43	44±9	28	70	50	48±15	27	85
**Landscape variables**								
Oak density ≥100 cm 5 km	1.5	2.4±2.3	0.5	10.9	1.6	2.0±1.5	0.4	6.2
(no. trees/km^2^)^a^								
Oak density ≥160 cm 0.5 km	0	0.6±0.9	0	2.5	0	0.9±2.8	0	12.7
(no. trees/km^2^)^b^								
Oak density ≥160 cm 2 km	0.1	0.2±0.4	0	2.0	0.2	0.3±0.6	0	2.8
(no. trees/km^2^)^c^								

a Included in the model for *Chaenotheca phaeocephala* occurrence.

b Included in the model for species richness, and in the models for *Cliostomum corrugatum*, *Buellia violaceofusca* and *Calicium adspersum* occurrences.

c Included in the model for *Ramalina baltica* occurrence.

### Effects of secondary woodland and canopy cover

The richness of the study species on ancient oaks in secondary woodland was on average 53% lower than on oaks in open conditions (Table 3). The multi-model also revealed that canopy cover did not explain species richness on oaks with bark crevices of low or intermediate depth, while the species richness decreased with increasing canopy cover on oaks with deep bark crevices (Table 3; an interaction plot not shown).

**Table 3:** Count regression models explaining species richness (an averaged model based on three plausible models) of red-listed epiphytic lichens on 52 ancient oaks.

Response variable	Parameter estimate^a,b^	Lower 95% CI*	Upper 95% CI*
Explanatory variables			
**Species richness**			
Intercept	0.0049	0.0038	0.0064
Bark pH	1.66	1.17	2.35
Secondary woodland	0.47	0.23	0.95
Max bark crevice depth (mm)	1.056	1.029	1.083
Canopy cover (%)	1.002	0.973	1.032
Bryophyte abundance (%)	1.005	0.991	1.018
Oaks >160 cm in diameter within 0.5 km	1.015	0.955	1.079
Max bark crevice depth: Canopy cover	0.945	0.904	0.987

*The parameter estimates and confidence limits of the models are back-transformed: estimated values express the proportional change in species abundance per unit increase in the explanatory variable. For example. 1.05 and 0.95 express 5% increase and 5% decrease, respectively, in species abundance per unit increase in the explanatory variable.

The occurrences of *C. corrugatum* and *R. baltica* were lower on oaks in secondary woodland than on oaks in open conditions (*C. corrugatum*: Table 4; *R. baltica*: Fishers exact test: no occurrences on oaks in secondary woodland and 7 occurrences on oaks in open conditions, p = 0.044). The abundance of the most frequent species, *C. phaeocephala,* was also lower on occupied oaks in secondary woodland than on occupied oaks in open conditions. Its occurrence decreased with increasing canopy cover (Table 4), although this was true only for trees with deep bark crevices, as judged by an interaction plot not shown. The abundance of *B. violaceofusca* also decreased with increasing canopy cover (Table 4). The occurrence and abundance of *C. adspersum* was better explained by environmental variables other than secondary woodland or canopy cover.

**Table 4:** Zero-inflated count regression models explaining abundance and non-occurrence of red-listed epiphytic lichens on 52 ancient oaks.

Type of model	Response variableExplanatory variables	Parameter estimate^a^	Lower 95% CI^a^	Upper 95% CI^a^	Test statistica (z-values) b	p^b^
	*Chaenotheca phaeocephala* (R^2^ = 0.417)					
Count	Intercept	0.105	0.080	0.138	−16.21	<0.001
	Secondary woodland	0.36	0.19	0.68	−3.10	0.002
	Bryophyte abundance (%)	0.98	0.96	1.00	−2.35	0.019
	Theta^c^	1.79	1.04	3.08	2.11	0.035
Binom^a^	Intercept	−1.65	−2.77	−0.53	−2.88	0.004
	Max bark crevice depth (mm)	−0.16	−0.28	−0.05	−2.77	0.006
	,Canopy cover	0.19	0.01	0.36	2.08	0.037
	Max bark crevice depth:Canopy cover	0.023	0.004	0.042	2.37	0.018
	*Cliostomum corrugatum* (R^2^ = 0.276)					
Count	Intercept	0.06	0.03	0.12	−7.26	<0.002
	Bryophyte abundance (%)	0.94	0.89	1.00	−1.94	0.053
	Theta^c^	1.12	0.39	3.19	0.21	0.830
Binom^a^	Intercept	1.93	0.64	3.23	2.92	0.003
	Max bark crevice depth (mm)	−0.15	−0.27	−0.03	−2.39	0.017
	Secondary woodland	3.49	0.42	6.55	2.23	0.026
	Bark pH	−1.91	−3.64	−0.18	−2.17	0.030
	*Buellia violaceofusca* (R^2^ = 0.231)					
Count	Intercept	0.05	0.03	0.08	−11.14	<0.002
	Canopy cover (%)	0.95	0.92	1.00	−2.17	0.030
	Theta^c^	1.42	0.45	4.44	0.60	0.550
Binom^a^	Intercept	1.98	0.83	3.12	3.38	0.001
	Max bark crevice depth (mm)	−0.14	−0.26	−0.03	−2.50	0.012
	Bark pH	−1.40	−2.90	0.10	1.64	0.068
	Secondary woodland	2.07	−0.41	4.56	−1.83	0.102
	*Calicium adspersum* (R^2^ = 0.501)					
Count	Intercept	0.04	0.02	0.07	−11.08	<0.002
	Max bark crevice depth (mm)	1.06	1.01	1.11	2.37	0.018
	Theta^c^	2.42	0.48	12.06	1.08	0.282
Binom^a^	Intercept	2.16	1.11	3.21	4.03	<0.001
	Bark pH	1.85	−0.09	3.8	1.87	0.062

a The probability of non-occurrence. Hence, the interpretation of the signs of the estimates is the opposite of typical binary models.

b z-values for non-occurrence models and associated p-values.

c Theta is a parameter of the negative binomial variance function [67].

### Effect of other local and landscape variables

The richness of the study lichens on ancient oaks increased by 66% per unit increase in bark pH (Table 3). The richness also increased with increasing maximum bark crevice depth, but a plot of the interaction (not shown) revealed that this effect was significant only for oaks with low canopy cover.

The occurrence of three species and the abundance of one species increased with increasing depth of bark crevices (Table 4). A plot (not shown) of the model for *C. phaeocephala* revealed that the effect of maximum bark crevice depth was significant only for oaks with low canopy cover. The occurrence of one species increased with increasing bark pH, and the abundance of two species decreased with increasing bryophyte abundance (Table 4). The density of ancient oaks in the surrounding landscape did not explain any of the response variables.

The other plausible models for individual species (Table S3) had similar parameter estimates and confidence limits for the variables that were included in the final models (Table 4).

## Discussion

The richness of the red-listed, epiphytic study lichens, the occurrences of two species, and the abundance of one species were all lower on ancient oaks in secondary woodland compared to oaks in open conditions. These findings suggest that the development of secondary woodland on abandoned oak wood pastures will lead to losses of species of conservation concern that are associated with ancient oaks.

### Effect of secondary woodland and canopy cover

In secondary woodland, the richness of the epiphytic study lichens was about half of that of oaks in open conditions. This pattern was also evident in individual species: the occurrences of two out of five species, and the abundance of one out of four species were significantly lower on oaks in secondary woodland compared to oaks in open conditions. We are not aware of other studies exploring the relationship between species richness, occurrence and abundance of red-listed epiphytic lichens and secondary woodland around ancient oaks. It is known that secondary woodland affects the species composition of epiphytic lichens [40], [41], but these studies do not report effects on species richness, occurrence or abundance of individual species, and do not separate the effects on threatened or other rare species.

A potential explanation for the lower levels of the study lichens in secondary woodland is that many of these stress-tolerant, mainly crustose, lichens are adapted to dry and light conditions [42] which are typical of oaks located in open conditions. During the development of secondary woodland around an ancient tree, the lichen colonisation and growth rate may decrease and the extinction rate may increase due to unsuitable conditions (e.g. too dark for photosynthesis or competition from foliose and fruticose lichens and bryophytes). Decreased wind speed may decrease the dispersal of diaspores that in turn decreases the lichen colonisation rate. Lõhmus and Lõhmus found a higher rate of colonisation of lichens on retention trees on clear cuts compared to forests [43]. It is also possible that large herbivores, which occur in open areas but not in woodlands, act as dispersal agents for epiphytic lichens, as they do for vascular plants [44]–[45].

Our results are in accordance with the findings of higher growth rate of two rare lichens on retention trees in small clear-cut areas and moderately thinned forests compared to in uncut controls [46]. However, our results contrast to the pattern of much lower abundance and fertility of two rare lichens on retention trees on clear-cuts compared to trees in closed forest [47]. Moreover, the growth rate of two red-listed epiphytic lichens was unaffected and the growth rate of one rare lichen decreased after selective cutting compared to lichens on trees in uncut stands [48]. The difference in forest structure in these studies may explain the difference in the lichen responses: moderate thinning in forests and the semi-open structure of wooded pastures, as in the current study, may be positive [46] (or neutral [48]) for many rare forest-dwelling lichens, while clear-cutting [47] may be negative or even detrimental.

Species richness, the occurrence of one species, and the abundance of another species decreased with increasing canopy cover (i.e. decreasing sun-exposure of the bark). Earlier studies of rare epiphytic lichens have not shown such relationships [30], [49]–[50]. The effect of canopy cover on species richness and occurrence were however, only important for oaks with deep bark crevices (i.e. for the oldest oaks, cf [50]). This may explain the lack of effect in the earlier studies, in which the average diameter of the trees were less than half of the average diameter in the current study, and in which no interactions were accounted for in the analyses. The richness of common epiphytic lichens however, either increases or decreases with increasing canopy cover, eg. [51]–[54]. The facts that the study lichens were negatively related to both secondary woodland and increasing canopy cover, and that earlier studies of rare species do not find any effect of canopy cover, suggests that factors related to secondary woodland other than current light conditions are important in explaining the richness of these species.

Regardless of the reasons for the responses by rare lichens to secondary woodland and canopy cover, conclusions about management for rare lichens should be based on the overall physical structure of the woodland, rather than on pure measurements of the current light conditions of single focal trees using canopy cover.

### Effect of other local and landscape variables

We found effects of the nuisance variables that were included in the modelling for refining the test of the effect of development of secondary woodland. The species richness and the probability of occurrence of the three most frequent of the study species increased with increasing bark crevice depth. This relationship has previously been found in common as well as rare epiphytes on different tree species [6], [42], [50], [53]. The suggested mechanisms behind the positive relationship is correlation with bark moisture and chemistry, with diaspore flush-off from the bark (less from rough than from smooth bark), with tree diameter (reflecting the score area for wind dispersed diaspores), or with age [6].

The species richness and the probability of occurrence of one red-listed species increased with increasing bark pH. This relationship has also previously been found, eg. [51], [53]–[55], but the current study shows that this is also true for red-listed epiphytes on oak.

The abundances of two study lichen species decreased with increasing bryophyte abundance, a frequently found correlation, e.g. [56], [50], [53]. It is known that lichen species compete in certain environments [57], and that bryophytes can reduce colonisation rates and increase extinction rates of epixylic lichens [58], but the relative importance of competition in structuring epiphyte communities is still unclear.

The distribution of the study species was not explained by the amount of their habitat (density of ancient oaks) in the surrounding landscape. This is surprising given landscape ecological and metapopulation theory, which assume increased colonisation rate with increasing connectivity between habitat patches [31], [32]. In addition, Paltto et al. [23] show increasing richness and occurrence of these epiphytic lichens with increasing density of surrounding ancient oaks. The lack of significant effects of landscape structure in the current study may be explained by the study design aiming to minimize the effects of landscape structure. The study oaks were located in a geographically small area resulting in considerably less variation in landscape structure. The sample size in the current study was also lower. The number of study oaks in open conditions was 31 and in Paltto et al. [23] it was 50. An additional reason may be that the oaks in secondary woodland are less suitable for the study species, leading to an even smaller effective sample size.

### Conclusions

Our study shows that the development of secondary woodland on oak wood pastures, which is an ongoing process in northern and western Europe, is most likely to lead to loss of lichen species that are now of conservation concern. In addition, oak experiences high mortality in closed canopy forests, cf [59], [60], which means that the ancient oaks themselves are also threatened by the development of secondary woodland. One exception to this may be oak forests in mountain areas, where the soils are thin and less suitable for most other tree species. However, the total area of such forests is negligible in the study region. We therefore recommend further management in existing oak pastures and restoration of secondary woodlands with ancient oaks. The most important management actions include clearing the young trees from around the oaks, and grazing near the ancient ones. The management plans should also include solitary oaks along roads, on farms and estates which are often overlooked.

Our management recommendations will benefit several additional organism groups. The development of secondary woodland on oak wood pastures is detrimental for a guild of beetles living in hollow oaks [12]. In addition, several species living in secondary woodland with oaks will benefit, or at least do not suffer, from restoration cutting in these environments, e.g. vascular plants, bryophytes, fungi, beetles, and mycetophilids [61]–[66]. The only group that seems to be negatively affected by such management is fungi within the phylum basidiomycetes [63].

## Supporting Information

Table S1 Characteristics of the ten red-listed study lichens.
(PDF)

Table S2 Raw data.
(TXT)

Table S3 Alternative models for the occurrence and abundance of individual species.
(PDF)

Table S4 The correlation between local variables and landscape variables.
(PDF)
