# Supplementary material for: Development of Secondary Woodland in Oak Wood Pastures Reduces the Richness of Rare Epiphytic Lichens
Source: PLoS One. 2011 Sep 22;6(9):e24675. doi: 10.1371/journal.pone.0024675 (PMC3178531; doi:10.1371/journal.pone.0024675)
Supplement: Table S1 — Characteristics of the ten red-listed study lichens. (PDF) [file pone.0024675.s001.pdf]

Table S1. Characteristics of the ten red-listed study lichens.

Species	Growth form	Reproductive/dispersal units*	Algal symbiont*	Habitat*	Range*
Chaenotheca phaeocephala (Turner) Th.Fr.	Crustose (calicioid)	Spores (6.5 µm)	Trebouxia	Bark and lignum of old trees, esp. oaks	Europe, N America, Russia, Japan
Cliostomum corrugatum (Ach.: Fr.) Fr.	Crustose	Spores (10 x 3 µm)/ conidia (2.5 x 1 µm)	Trebouxia	Bark of old oaks, lignum	Europe, N America
Ramalina baltica Lettau	Foliose	Soredia (10 x 6 µm)	Trebouxia	Bark of old trees, esp. oaks	Europe, N America
Buellia violaceofusca G. Thor & Muhr	Crustose	Soredia (18 µm)	Trebouxia	Bark of old trees, esp. oaks	Europe, N America
Calicium adspersum Pers.	Crustose (calicioid)	Spores (15 x 7 µm/ conidia (2.5 x 1.3 µm)	Trebouxia	Bark of old oaks	Europe, New Zealand
Calicium quercinum Pers.	Crustose (calicioid)	Spores (11.5 x 5.5 µm)	Trebouxia	Bark and lignum of old oaks	Europe
Sclerophora coniophaea (Norman) J. Mattsson & Middelb.	Crustose (calicioid)	Spores (5 µm)	Trentepohlia	Bark and lignum of oaks (*Betula*, *Picea* in e.g. N Sweden)	Europe, N America, Asia
Schismatomma pericleum (Ach.) Branth & Rostr.	Crustose	Spores (34 x 3.3 µm/soredia (-30 µm)	Trentepohlia	Bark of oaks (*Picea* in e.g. N Sweden)	Europe
Caloplaca lucifuga G. Thor	Crustose	Soredia (21 µm)	Trebouxia	Bark of old trees, esp. oaks	W Europe
Lecanographa amylacea (Ehrh. Ex Pers.) Egea & Torrente	Crustose	Spores (22 x 3.3 µm)	Trentepohlia	Bark of old oaks	Europe, N America

* Obtained from:

- Thor G (1988) *Caloplaca lucifuga*: A new lichen species from Europe. Lichenologist 20: 175-178
- Purvis O W, Coppins B J, Hawksworth D L, James P W, Moore D M (eds) (1992) The lichen flora of Great Britain and Ireland. Natural History Museum Publications, London, UK
- Lorentsson S (1997) Differences between *Ramalina baltica* and *Ramalina obtusata*. [In Swedish: with English summary] Svensk Bot Tidskr 91:591-598
- Thor G, Arvidsson L (eds) (1999) Swedish red data book on lichens. ArtDatabanken, Swedish University of Agricultural Sciences, Uppsala
- Foucard T (2001) Svenska skorplavar och svampar som växer på dem [Crustose lichens and fungi associated with these]. Interpublishing, Stockholm, Sweden
