# Supplementary material for: Development of Secondary Woodland in Oak Wood Pastures Reduces the Richness of Rare Epiphytic Lichens
Source: PLoS One. 2011 Sep 22;6(9):e24675. doi: 10.1371/journal.pone.0024675 (PMC3178531; doi:10.1371/journal.pone.0024675)
Supplement: Table S3 — Alternative models for the occurrence and abundance of individual species. (PDF) [file pone.0024675.s003.pdf]

Table S3. Alternative models for abundance and occurrence of individual species. Models presented in Table 3, and alternative models with delta-AIC < 2.0. Akaike weights, parameter estimates with confidence limits, z-statistics and associated p-values.

Akaike weights for models

	Model presented in Table 3	Alt. Model 1	Alt. Model 2	Alt. Model 3
Cliostomum corrugatum	0.71	0.29		
Buellia violaceofusca	0.41	0.23	0.19	0.17
Calicium adspersum	0.37	0.34	0.29	

Chaenotheca phaecephala

No alternative models with delta-AIC < 2.0

Cliostomum corrugatum - model results

Model presented in Table 3 (AIC_c = 168.2, R^2 =0.276):

	Estimate	Lower 95 % CI	Upper 95 % CI	z	p
Count model (abundance)					
Intercept	0.06	0.03	0.12	-7.26	<0.002
Bryophyte abundance (%)	0.94	0.89	1	-1.94	0.053
Theta	1.12	0.39	3.19	0.21	0.83
Binomial model (non-occurrence)					
Intercept	1.93	0.64	3.23	2.92	0.003
Bark pH	-1.91	-3.64	-0.18	-2.17	0.030
Max bark crevice depth (mm)	-0.15	-0.26	-0.03	-2.39	0.017
Secondary woodland (absence 0, presence 1)	3.49	0.42	6.55	2.23	0.026

Alternative model 1 (AIC_c = 170.0, R^2 = 0.223) (canopy cover instead of bryophyte abundance in the count sub-model)

	Estimate	Lower 95 % CI	Upper 95 % CI	z	p
Count model (abundance)					
Intercept	0.1	0.06	0.18	-7.77	<0.002
Canopy cover (%)	0.97	0.93	1.01	-1.33	0.184

Theta	0.97	0.32	2.99	-0.05	0.961
Binomial model (non-occurrence)					
Intercept	2.09	0.66	3.52	2.86	0.004
Bark pH	-1.96	-3.77	-0.16	-2.13	0.033
Max bark crevice depth (mm)	-0.16	-0.32	0	-1.95	0.051
Secondary woodland (absence 0, presence 1)	3.89	0.05	7.72	1.99	0.047

Buellia violaceofusca - model results

Model presented in Table 3 (AIC_c = 134.0, R^2 = 0.231):

	Estimate	Lower 95 % CI	Upper 95 % CI	z	p
Count model (abundance)					
Intercept	0.05	0.03	0.08	-11.14	<0.002
Canopy cover (%)	0.95	0.92	1	-2.17	0.030
Theta	1.42	0.45	4.44	0.6	0.550
Binomial model (non-occurrence)					
Intercept	1.98	0.83	3.12	3.38	0.001
Max bark crevice depth (mm)	-0.14	-0.26	-0.03	-2.5	0.012
Secondary woodland (absence 0, presence 1)	2.07	-0.41	4.56	1.64	0.102
Bark pH	-1.4	-2.9	0.1	-1.83	0.068

Alternative model 1 - AICc = 135.1, R^2 = 0.167) (secondary woodland omitted from the non-occurrence sub-model)

	Estimate	Lower 95 % CI	Upper 95 % CI	z	p
Count model (abundance)					
Intercept	0.047	0.027	0.081	-10.96	<0.002
Canopy cover (%)	0.953	0.913	0.995	-2.21	0.027
Theta	1.38	0.43	4.41	0.54	0.590
Binomial model (non-occurrence)					
Intercept	1.6	0.71	2.48	3.53	<0.001
Max bark crevice depth (mm)	-0.11	-0.19	-0.02	-2.55	0.011
Bark pH	-1.3	-2.65	0.05	-1.88	0.060

Alternative model 2 - AIC_c = 135.5, R^2 = 0.176) (bark pH omitted from the non-occurrence sub-model)

	Estimate	Lower 95 % CI	Upper 95 % CI	z	p
Count model (abundance)					
Intercept	0.05	0.03	0.08	-10.99	<0.002
Canopy cover (%)	0.95	0.92	1	-2.15	0.032
Theta	1.39	0.42	4.52	0.54	0.589
Binomial model (non-occurrence)					
Intercept	1.75	0.76	2.74	3.46	0.001
Max bark crevice depth (mm)	-0.12	-0.22	-0.02	-2.4	0.016
Secondary woodland (absence 0, presence 1)	1.93	-0.41	4.27	1.62	0.106

Alternative model 3 (AIC_c = 135.8, R^2 =0.194) (canopy cover instead of secondary woodland in the non-occurrence sub-model)

	Estimate	Lower 95 % CI	Upper 95 % CI	z	p
Count model (abundance)					
Intercept	0.048	0.029	0.081	-11.46	<0.002
Canopy cover (%)	0.96	0.92	1	-2.19	0.029
Theta	1.49	0.51	4.37	0.73	0.466
Binomial model (non-occurrence)					
Intercept	1.79	0.79	2.8	3.51	<0.001
Max bark crevice depth (mm)	-0.13	-0.22	-0.04	-2.84	0.005
Canopy cover (%)	0.06	-0.03	0.14	1.36	0.174
Bark pH	-1.45	-2.9	<0.01	-1.95	0.051

Ramalina baltica

No models significant

Calicium adspersum

Model presented in Table 3 (AIC_c = 96.5, R^2 = 0.501):

	Estimate	Lower 95 % CI	Upper 95 % CI	z	p
Count model (abundance)					
Intercept	0.04	0.02	0.07	-11.08	<0.002
Max bark crevice depth (mm)	1.06	1.01	1.11	2.37	0.018

Theta	2.42	0.48	12.06	1.08	0.282
Binomial model (non-occurrence)					
Intercept	2.16	1.11	3.21	4.03	<0.001
Bark pH	1.85	-0.09	3.8	1.87	0.062
**Alternative model 1 - AIC_c = 96.6, R^2 = 0.352) (Oak density in the landscape added to the non-occurrence sub-model)**					
	**Estimate**	**Lower 95 % CI**	**Upper 95 % CI**	**z**	**p**
Count model (abundance)					
Intercept	0.037	0.021	0.065	-11.18	<0.002
Max bark crevice depth (mm)	1.06	1.01	1.11	2.39	0.017
Theta	2.45	0.5	11.98	1.11	0.2671
Binomial model (non-occurrence)					
Intercept	2.14	1.11	3.18	4.05	<0.001
Bark pH	1.49	-0.42	3.4	1.53	0.125
Oaks >160 cm in diameter within 0.5 km	-0.31	-0.83	0.22	-1.15	0.251
**Alternative model 2 - AIC_c = 97.0, R^2 = 0.196) (Oak density in the landscape added and bark pH omitted from the non-occurrence sub-model)**					
	**Estimate**	**Lower 95 % CI**	**Upper 95 % CI**	**z**	**p**
Count model (abundance)					
Intercept	0.037	0.02	0.066	-11.12	<0.002
Max bark crevice depth (mm)	1.06	1.01	1.11	2.38	0.017
Theta	2.43	0.5	11.97	1.1	0.274
Binomial model (non-occurrence)					
Intercept	1.92	1.06	2.78	4.36	<0.001
Oaks >160 cm in diameter within 0.5 km	-0.38	-0.91	0.15	-1.41	0.159
