# Supplementary material for: Development of Secondary Woodland in Oak Wood Pastures Reduces the Richness of Rare Epiphytic Lichens
Source: PLoS One. 2011 Sep 22;6(9):e24675. doi: 10.1371/journal.pone.0024675 (PMC3178531; doi:10.1371/journal.pone.0024675)
Supplement: Table S4 — The correlation between local variables and landscape variables. (PDF) [file pone.0024675.s004.pdf]

Table S4. The correlation between local variables and landscape variables. Test statistica and significance level from pairwise Spearman rank correlation tests.

	Canopy cover	Bryophyte abundance	Crevice depth	bark pH	Oaks >100 cm 5 km	Oaks >160 cm 0.5 km	Oaks >160 cm 2 km
Secondary woodland (absence=0, presence=1)	0.65***	0.40**	0.11	-0.06	-0.02	-0.11	0.17
Canopy cover (%)		0.32*	0.24	0.04	-0.08	-0.01	0.11
Bryophyte abundance (%)			-0.12	0.04	-0.04	-0.07	0.08
Maximum bark crevice depth				-0.16	0.25	0.01	0.07
Bark pH					0.06	-0.05	0.07

* $0.01 < p < 0.05$, ** $0.001 < p < 0.01$, *** $p<0.001$
